# Examining Waiting Placement in Hospital: Utilization and the Lived Experience

**DOI:** 10.5539/gjhs.v6n2p12

**Published:** 2013-11-14

**Authors:** Donna M. Wilson, Jill Vihos, Jessica A. Hewitt, Nancy Barnes, Karen Peterson, Ralph Magnus

**Affiliations:** 1 Faculty of Nursing, University of Alberta, Edmonton, Canada; 2 Chaplain Services, Covenant Health, Grey Nuns Hospital, Edmonton, Canada

**Keywords:** waiting placement, alternative level of care, nursing home, hospital utilization, phenomenology, research

## Abstract

This mixed-methods study addressed the problem that although waiting placement is considered a major hospital utilization issue, minimal evidence exists to highlight the extent of it and the personal impact of waiting placement. An analysis of two years of complete hospital data for the Canadian province of Alberta was undertaken to examine waiting placement rates and describe waiting placement patients. Qualitative interviews and observations of elderly patients waiting in hospital for nursing home placement were also undertaken to gain an understanding of the lived experience of waiting for placement in hospital. Only 1.8% of all inpatients were waiting placement with an ALC (Alternative Level of Care) designation, 80% of ALC waits were less than 41 days (mean=29.85, median=14), and 2.2% of total hospital bed days in these two years were used by ALC patients. Three qualitative themes emerged: (a) coming to a realization of this significant move, (b) waiting is boring and distressing, and (c) hospitals are not designed for waiting placement. The findings of this study should raise awareness that although relatively few people wait placement in hospital, there are some major possible negative effects of waiting for placement in hospital for those who wait; with remedies to address waiting placement care deficits needed.

## 1. Introduction

Hospitals are a common transition point for nursing home entry, as only around 40% of the people who move into nursing homes are admitted directly from the community (Canadian Institutes for Health Information [CIHI], 2009; Devroey, Van Casteren, & De Lepeleire, 2002; Goodwin, Howrey, Zhang, & Kuo, 2011). Transfers from hospitals to nursing homes typically occur after a determination has been made that the person’s care needs exceed family and/or community resources, often because they require substantial ongoing assistance with activities of daily living (Carey et al., 2008; CIHI; Meiland et al., 2001; Penrod & Dellasega, 1998; Sheps et al., 2000). Although younger people may need institutionally-based supportive care, frail-elderly people are much more likely candidates for nursing homes, as physical illnesses and/or cognitive disabilities are typically more common, pronounced, and problematic in advanced old age (Wilson & Truman, 2004). With population aging beginning to accelerate now, many more elderly people in the future could wait in hospital for a nursing home bed. These waits can be lengthy (CIHI; Epure, & Blanchette, 2002; Mayo, Wood-Dauphinee, Gayton, & Scott, 1997), and this use of expensive and scarce hospital beds has been of considerable concern for some time now in Canada and many other developed countries (Beland et al., 2006; CIHI, Devroey et al.; Fjelltun, Henriksen, Norberg, Gilje, & Normann, 2009; Murtaugh & Litke, 2002).

Long hospital stays also pose some serious hazards for older patients, including an enhanced risk of acquiring a nosocomial infection and experiencing other adverse events in hospital (Baker et al., 2004). Long hospital stays have been linked with depression and cognitive deterioration for older patients, as well as physical deconditioning and functional decline (Fjelltun et al., 2009; Mayo et al., 1997; Penrod & Dellasega, 1998). Yet, surprisingly, few studies have focused on waiting placement. One relatively recent online report highlighted the proportion of hospital patients in Canada who wait placement and their characteristics; with waiting placement or alternative level of care (ALC) days ranged from 1 to 100 days in length across Canada, and in the 2007-08 year, 5% of all hospital admissions and 14% of total hospital days involved ALC patients (CIHI, 2009). ALC patients were identified as “older” and more likely to begin their hospital experience in a hospital emergency department as compared to non-ALC patients (CIHI, 2009). Dementia was a common primary diagnosis or co-morbidity for ALC patients (CIHI, 2009). No studies appear to have sought an understanding of the impact of this highly significant wait on the people who find they are waiting in hospital for a nursing home bed. A two-part mixed methods study was undertaken to gain an understanding of the lived experience of older patients as they wait in hospital for a nursing home bed, describe waiting placement patients and clarify their share of hospital utilization in Alberta, a western-Canadian province.

## 2. Methods

Before initiating this study, research ethics approval was obtained from a University of Alberta Health Research Ethics Board. The two phases were initiated concurrently.

### 2.1 Quantitative Hospital Utilization Phase

Alberta Health (the government department responsible for health services and health data) was asked to provide the two most recent years of complete inpatient hospital (i.e. Discharge Abstracts Database or DAD) data for the province for a secondary data analysis. Select data are routinely collected on all patients in every hospital across the province of Alberta. Around 6,800 hospital beds exist in this province of 4 million persons, with 10.4% of provincial residents in 2009 aged 65 or older (Government of Alberta, 2010). In 2010, data for the years 2006-07 and 2007-08 were received following their administrative approval of this study and after a data-retrieval fee was paid. Each dataset was defined by fiscal year, starting April 1 through March 31. For every person appearing in these datasets, matching individual-anonymous socio-demographic data from a Registry database were provided. Every individual had a unique number assigned by Alberta Health that was common across the datasets. This number permitted an individual analysis of hospital utilization over multiple admissions and combined years. The data were examined after being uploaded into the Microsoft ACCESS program. Minimal missing data (<2%) for each variable were noted.

Two data years were requested to enable an assessment of data quality through a year to year comparison of findings. Few patient and utilization differences were noted across the two years. In the first year, there were 275,738 separations from hospital either as a live or deceased person, and 281,320 the second year. Data were explored using the frequencies and summary statistics functions of the SPSS computer program (Version 18); including means, medians, modes, standard deviations, and ranges. Similarities and differences within and across each year were noted, with data each year and across the two years combined then explored using relevant statistical tests to compare mean scores and distribution probabilities, and assess for relationships between variables.

### 2.2 Qualitative Phase

The qualitative study phase was undertaken in the same year (2010) that the quantitative data were received to answer the question: What is the lived experience of elderly hospital in-patients as they wait in hospital for placement in a nursing home? Phenomenology was the chosen method, as phenomenology was designed to gain an understanding of “lived” experiences (Flood, 2010). Phenomenology is a commonly used qualitative research method, in part because it addresses the issue that people have difficulty understanding an event or process if they have never experienced it. They may not then realize that the event or process is highly impactful on other people. Phenomenology is understood as providing a voice to population groups that are silent, unheard, or unnoticed (Riley & Manias, 2004). Phenomenology typically involves interviewing key informants until data saturation - a point when no new information or new understandings about the collected data emerge (Pope & Mays, 2006). Phenomenology is expected to generate new insights and information about real life issues or matters (Flood, 2010).

Our qualitative data gathering involved interviewing older (age 65+), English-speaking, mentally-competent patients at two full-service hospitals in Alberta. Only older persons were interviewed, as although younger persons wait placement at times, the reasons younger persons wait placement could differ from those of senior citizens – the most common nursing home resident (Wilson & Truman, 2004). One interview was planned for each hospital patient who met the criteria for this study and voluntarily participated in an interview. The decision to not do serial interviews was made because of the anticipated ill health of the participants and the potential for their transfer to a nursing home before a second interview could take place. Data gathering in nursing homes was not undertaken as this study phase focused on the wait in hospital and memory or recall issues could distort findings. To gain participants, potentially participants were identified each week by the nurse managers on all medical and surgical wards of the two hospitals. Potential participants had to have been designated as an ALC patient, and were considered competent by the nurses to make decisions and give consent. All patients who volunteered for this study after receiving written information about the study from a unit nurse, and then after receiving in-depth written and verbal information from a research assistant were interviewed. All participants signed a consent form before data gathering began.

As the immediate setting and broader context are considered important for high-quality qualitative research (Flood, 2010), extensive field notes were written to record details about each participant and other observations. Photographs were taken of each participant’s hospital room at the end of the interview if they consented to this, since photovoice provides additional insights about “invisible” people or places (Catalani & Minkler, 2010). Care was taken to ensure these photographs did not identify any patient or the hospital. A semi-structured interview guide containing five open-ended questions was the primary tool to collect data. This interview guide helped ensure data-gathering consistency over time and across two research assistants (one assigned to each hospital). Both assistants were experienced hospital nurses who were undertaking a Master’s in Nursing program, and each was trained by the Principal Investigator on the collection and recording of qualitative data. The questions were:

1) What happened to you - what made you come into hospital?
2) How did you hear or learn that you would be moving to a nursing home?
3) How do you feel about going to a nursing home?
4) What do you do each day, now that you are waiting for a nursing home bed?
5) What is it like to be waiting in hospital for placement in a nursing home?

A purposive sample of 20-25 participants was originally planned, as a larger number of participants is recommended when repeat interviews are not possible (Richards & Morse, 2007). Gaining 20-25 participants was not thought problematic, as waiting placement has often been cited as the main reason why hospitals are full in Canada (CIHI, 2009). However, 12 months passed until nine suitable volunteers were recruited from approximately 200 candidates and data saturation occurred. During these 12 months, most (90%) waiting placement or ALC patients could not be approached as they were actively dying or had major cognitive deficits. Among the remaining, approximately half declined to participate, with these typically indicating that they did not want to think about or talk about waiting for placement and their eventual move into a nursing home.

Data analysis consisted of a manual analysis of transcribed interview data and a concurrent review of the patient’s other textual and photographic information soon after data collection. Data from each subsequent patient were then compared with the data gained from previous patients, a data analysis method common to grounded theory research (Richards & Morse, 2007). This constant-comparative data analysis method was employed because a month or more passed between interviews, and it was important for the Principal Investigator and the research assistants to remain familiar with the data and the emerging themes, and to plan for data refinement at future interviews. Data analysis consisted of coding key findings and then grouping these into categories; with these categories then grouped into themes. Representative quotes, photographs, and field notations were selected for illustration purposes.

## 3. Results

### 3.1 Quantitative Phase

Table 1 shows 2.3% of all persons admitted to an inpatient hospital bed in these two years had an ALC designation. In most cases, the ALC designation was made after hospital admission, as only 221 (or 1.7%) of the 13,043 patients who had an ALC designation were admitted from a nursing home with this designation. ALC patients were older on average as compared to other patients, with 85.3% of all ALC patients 65+ years of age as compared to 23.6% of all other patients. Among the ALC patients, 80% of their ALC waits were less than 41 days in length, with the mean wait after an ALC designation of 29.85 days (median=14, mode=7). When the pre-ALC days and ALC days were totaled for each ALC patient, their entire hospital stay averaged 72.0 days (median=49, mode=37), as compared to 7.8 days for all other patients. Although this entire hospital stay was nearly 10 times longer on average, patients with ALC designations were only accountable for 2.2% of total hospital bed days accumulated in the province over these two years combined.

**Table 1:** ALC and other hospital patients (2006-08), comparative findings

	2006-07 Year	2007-08 Year	Two Years Combined
Number	275,738 (100%)	281,320 (100%)	557,058 (100%)
- ALC Designation	6,596 (2.4%)	6447 (2.3%)	13,043 (2.3%)
- Non-ALC/Other	269,142 (97.6%)	274,873 (97.7%)	544,015 (97.7%)
Predominant Gender			
- ALC Designation	Female (59.0%)	Female (58.5%)	Female (58.8%)
- Non-ALC/Other	Female (59.6%)	Female (60.0%)	Female (59.8%)
Average Age (years)			
- ALC designation	77.3	77.5	77.4
- Non-ALC/Other	39.75	39.2	39.5
Average Diagnoses			
- ALC designation	10.1	10.5	10.3
- Non-ALC/Other	4.0	4.0	4.0
Primary Diagnosis (%)			
- ALC designation	Factors Influencing Health Status and Contact with Health System (22.9%)	Factors Influencing Health Status and Contact with Health System (22.5%)	Factors Influencing Health Status and Contact with Health System (22.7%)
- Non-ALC/Other	Factors Influencing Health Status and Contact with Health System (16.5%)	Factors Influencing Health Status and Contact with Health System (17.1%)	Factors Influencing Health Status and Contact with Health System (16. 8%)
Average Procedures			
- ALC designation	0.9	0.9	0.9
- Non-ALC/Other	1.3	1.3	1.3
Special Care Unit (%)			
- ALC designation	6.9%	5.9%	6.3%
- Non-ALC/Other	8.9%	8.5%	8.7%
Mean ALC Days			
- ALC designation	25.4	34.4	29.85
Mean Days in Hospital			
- ALC designation	66.4	77.7	72.0
- Non-ALC/Other	7.7	7.8	7.8

ALC patients varied considerably (see Tables 1, 2 and 3); ranging in age from 0-106, with 14.7% under age 65 and 53.7% aged 81+ years. Over half (58.8%) were female. A wide range of diagnosed health problems were evident, with the most common primary diagnosis or ICD code “factors influencing health status and contact with health system” (22.7%), followed by a “mental or behavioural disorder” (12.8%), and a circulatory disease (12.0%). ALC patients had 1 to 25 recorded diagnoses each. Almost all had multiple co-morbidities, with 10.9 the average number. Despite this, 65.0% had no surgery or major diagnostic tests performed in hospital; 0.9 was the average number of procedures performed per ALC patient. Only 6.4% of the ALC patients received care in a special care unit, such as a coronary care or intensive care unit. Among all 13,043 ALC patients, 900 (6.9%) died in hospital, 1,542 (11.8%) were transferred to another hospital (rehabilitation or small rural usually), 2,699 (20.7%) were discharged home, and 60.6% (n=7,902) were transferred to a nursing home.

**Table 2:** ALC patients differentiated by those who were transferred to a nursing home or not

Two Years (2006-07 and 2007-08) Combined (N=13,043)	Transferred to a Nursing Home	Not Transferred to a Nursing Home	All ALC Patients
Number (%)	7,902 (60.6%)	5,141 (39.4%)	13,043 (100%)
Predominant Gender (%)	Female (60.3%)	Female (56.4%)	Female (59.8%)
Average Age (years)	80.2	73.1	77.4
Average Diagnoses (number)	10.7	9.7	10.3
Most Common Diagnosis (%)	Factors Influencing Health Status and Contact with Health System (18.8%)	Factors Influencing Health Status and Contact with Health System (28.7%)	Factors Influencing Health Status and Contact with Health System (22.7%)
Second Most Common Diagnosis (%)	Mental or Behavioural Disorder (13.6%)	Mental or Behavioural Disorder (11.6%)	Mental or Behavioural Disorder (12.8%)
Third Most Common Diagnosis (%)	Circulatory Disorders (13.2%)	Circulatory Disorders (10.3%)	Circulatory Disorders (12.0%)
Fourth Most Common Diagnosis (%)	Injury, Poisoning, and Other Consequences of External Causes (11.4%)	Neoplasms (9.3%)	Injury, Poisoning, and Other Consequences of External Causes (10.1%)
Mean Procedures	.93	.91	0.93
Had Special Care Unit Care (%)	6.1%	6.6%	6.3%
Mean ALC Days	29.4	30.52	29.85
Mean Total Hospital Days	71.1	73.3	71.97

**Table 3:** ALC Patients Differentiated by Age (Younger and Older)

Two Years Combined (2006-07 and 2007-08)	Older (Age 65+)	Younger (<65)	All ALC Patients
Number (%)	10,909 (85.3%)	1,887 (14.7%)	12,796 (100%)
Predominant Gender (%)	Female (60.8%)	Male (53.2%)	Female (59.8%)
Mean Age (years)	82.7	46.9	77.4
Mean Diagnoses (number)	10.5	9.5	10.3
Most Common Diagnosis (%)	Factors Influencing Health Status and Contact with Health System (21.5%)	Factors Influencing Health Status and Contact with Health System (29.3%)	Factors Influencing Health Status and Contact with Health System (22.6%)
Second Most Common Diagnosis (%)	Mental or Behavioural Disorder (12.7%)	Injury, Poisoning, and Other Consequences of External Causes (13.9%)	Mental or Behavioural Disorder (12.8%)
Third Most Common Diagnosis (%)	Circulatory Disorders (12.5%)	Mental or Behavioural Disorder (13.6%)	Circulatory Disorders (12.0%)
Fourth Most Common Diagnosis (%)	Injury, Poisoning, and Other Consequences of External Causes (9.2%)	Circulatory Disorders (9.2%)	Injury, Poisoning, and Other Consequences of External Causes (10.1%)
Mean Procedures Done	.7	1.9	0.91
Had Special Care Unit Care	4.4%	17.2%	6.3%
Mean ALC Days	27.5	43.7	29.9
Mean Total Hospital Days	65.9	108.8	72.2

Table 2 shows ALC patients who were transferred to a nursing home differed minimally from those not transferred to a nursing home. The only statistically significant difference was age; ALC patients transferred to a nursing home were 7.1 years older on average. Table 3 shows ALC patients differed considerably when those aged 65+ were compared to those aged 0-64. Despite older ALC patients having more diagnoses on average, they were much less likely to be admitted to a special care unit and to have surgery or major diagnostic tests performed. Older ALC patients had a shorter ALC stay and shorter total hospital stay as compared to younger ALC patients.

### 3.2 Qualitative Phase

The nine interviewed participants ranged in age from 80 to 92 years old (median = 85). Six were female and three male. All interviews were conducted in their hospital room, the site chosen by them and acceptable to the research assistants for privacy and confidentiality purposes. Most interviews were under 30 minutes in length, as there were only a few easily understood questions, and the informants had little to report or discuss despite considerable efforts to encourage longer discussions. The interview data quickly became repetitive as the participants gave similar answers to the questions. Three themes, each with two sub-themes, rapidly emerged and were validated through subsequent interviews and the ongoing data analysis: (a) coming to a realization of this significant move, (b) waiting is boring and distressing, and (c) hospitals are not designed for waiting placement.

#### 3.2.1 Theme 1. Coming to a Realization of This Significant Move

Not only were the nine participants aware of the impending move to a nursing home and resigned to it, but they were also typically the recipient of this decision to move and rarely the decision-maker for it.

Sub-theme: Realization and resignation. All had come to realize they were being moved to a nursing home; they would not be returning to their previous home. They appeared resigned to this move because they had no other options, and most seemed aware that this move to a nursing home would be permanent because of their poor health and low physical functioning. One woman, for instance, shrugged and said “what can I do?” (G3). She had not regained her ability to walk during this hospitalization. Many expressed grief over multiple losses associated with this move, however. One man cried as he talked about his house where he had raised his children, saying that he was not going back to his “home” (G1). Another said she was an independent person and did not feel good about going to a nursing home as she would be “losing my independence and the freedom to make my own decisions” (M1).

Sub-theme: Decision-making involvement. Most were not consulted in the placement decision-making process, but were simply told by a family member or their doctor that they would be placed in a nursing home. One woman overheard her daughter say to her doctor, “she should go into a nursing home” (G2). This upset her, but mainly because she was “lonely” in the hospital and she wanted to “go home now.” She had not been lonely in her home, but that was when she had been able to care for herself independently. Another woman did not remember discussing this move with anyone and she “guessed” that the nurses had spoken to her about this move and that is how she came to know she was moving to a nursing home (M4). Moving to a nursing home was acceptable to her because she had once worked in a nursing home and she felt she would be “looked after there.” She still wished, however, that she had been given the opportunity to approve this move. One other woman, who had participated jointly with her family in making the decision for her to move into a nursing home (G3), indicated she looked forward to the nursing home. She said, “I and my family decided this would be best for me; I hope the move is soon.”

#### 3.2.2 Theme 2. Waits Are Boring and Distressing

The wait in hospital for the nursing home transfer was boring and distressing; with two sub-themes evident: (a) waiting for placement largely involved waiting and more waiting, and (b) these waits contributed to or resulted in loneliness and social isolation.

Sub-theme: Waiting and more waiting. All participants indicated that their daily routines consisted primarily of waiting. One male just said “waiting” and sighed when asked what he did each day (G1). He said he sometimes sits in his wheelchair in the hallway and hopes that a volunteer will stop by and take him outside for a while to pass the day. A few indicated that they were used to being busy each day at home, but now had nothing to do but wait. The field notes reflected sadness and frustration as they discussed having to wait for everything. A few became teary. When one woman was asked what she did each day, she replied, “What can I tell ya? Waiting, waiting, waiting… I don’t care for it” (M4). She looked down and wiped tears from her eyes as she said this. Many reported sleeping throughout the day because they had nothing else to do besides watch the clock. Some read books to help pass the time.

Sub-theme: Loneliness and social isolation. The experience of loneliness and/or social isolation was also revealed. One woman (G2) directly reported that she felt “lonely” in the hospital. She had few visitors, as all her friends were elderly and her children lived far away. One male (G1) openly expressed frustration at his separation from his wife, who was wheelchair bound and only able to visit him every few weeks. He was frustrated as he could do “nothing about it.” Another, when asked what she does every day, responded: “Nothing. I can’t start anything because I can’t finish anything” (M1). She said did not know when she would be moved to a nursing home, so she did not feel like starting to read a book or doing a crossword puzzle as these belonged to the hospital and she knew she could not take them with her when she moved. She did not attempt to make friends with other patients as she did not know how long she would be in contact with them. She also felt she had little to say to them as she knew she was going to a nursing home and they were not. No participant had been given a moving date and only one knew which nursing home he would be moved to.

Loneliness or social isolation was also revealed in other ways. One male, who was lying in a wet bed, said he did not know when the nurses would be able to come to help him (G1). He had been in hospital for seven weeks and although he reported that the nurses were “kind” to him, he said he had to be patient while waiting for help. He was not able to get out of bed on his own, and he was in a room with no other patients. Other than the brief episodes when one or more nurses were in his room providing care to him, he said he was “alone the rest of the day.” Most indicated they rarely saw anyone other than hospital staff each day, as few friends or family members visited. Many were cut off from their past social contacts through being in hospital, as the hospital was a considerable distance from their home or home community, with visitors often said to be unable to visit because of this distance. Only two had a bedside television to watch and one had a bedside telephone. The telephone did not ring during her interview. One woman said that she slept most of the time as she was “very bored being alone everyday” (G3). She reported that she felt “cut off” in the hospital from “everyone and everything familiar.”

#### 3.2.3 Theme 3. Hospitals Are Not Designed for Waiting Placement

The interviews and observations at these two acute care hospitals revealed few services or programs exist for waiting placement patients. This care gap contributed to physical and mental stagnation.

Sub-theme: Few services or programs. Most participants commented directly or indirectly that hospitals have few programs or services for waiting placement patients. The participant who had worked in a nursing home previously stated “too bad there are no services here like those in nursing homes” (M4). She knew of daily card games, bingo nights, group meals, organized bus trips to attend community events, and in-house pets that resided in nursing homes. One male who had been in the hospital for eight weeks said “they haven’t really done anything to service me here, just pills; that’s all, and nothing else” (M3). He wanted to move as soon as possible to a nursing home because he thought he would get more attention and personal care there. One female was also beginning to look forward to moving because she had been in hospital for two months and she thought nursing homes have “people that can help you out with everything that you need to do during the day” (G3). She also thought the nursing home would have “activities,” and so she would be able to do more than just watch the clock to “pass time,” as she did now. One daughter of a waiting placement participant later contacted the Principal Investigator to say that “it is unconscionable what they do to older people in hospital, there is nothing for her there; the doctors and nurses are not even interested in caring for Mom anymore now that she is not seriously ill, and yet I have to pay for her upkeep here.”

The field notes reflecting discussions with the hospital nurses about these patients also illustrated that few if any hospital programs or services exist to meet their unique needs. For instance, bedside televisions and telephones involved a substantial out-of-pocket daily fee ($15 and $5 per day respectively). These private fees are in addition to the daily accommodation fee that each is charged immediately after being designated as waiting placement. The appended pictures illustrate a standard hospital room with no personalization of it. Each room consisted of a hospital bed with hospital-issued linens, a bedside chair, two small tables, and with no personal items in view, such as family photographs. The field notes reflected all were wearing hospital-issue pajamas.

Sub-theme: Physical and mental stagnation. Physical and mental stagnation were visibly evident and apparent through the interviews. One male (M6) said he smoked cigarettes to “pass the time,” and his walk to the hospital entrance each time he wanted a cigarette was his only exercise each day. He said he was breathing heavier and it was getting harder for him to get around independently now. He said he was “weak because of a lack of exercise” in hospital. He alone could walk unaided; all others were bedbound and required 1- or 2-person ambulation assistance. All but one had been able to walk and care for their homes independently or with some help for major tasks before this hospitalization. One female expressed concern about her deteriorating strength as she said: “I sit on my butt most of the day. I want to get up and walk with a walker” (G2). She reported the nurses came when she called them, but she wanted more exercise to build her strength and be able to do more for herself but “because the hospital does not have enough staff, this is not possible” (G2).

Mental stagnation was also evidenced by the short interviews; the field notes indicated that all participants had little to say. Efforts to engage the participants in conversation on the topic of waiting placement and on other topics were not fruitful. Many simply repeated what they had said earlier and with little elaboration.

## 4. Discussion

The provincial hospital utilization data provides clear evidence that waiting for placement in hospital is not a common occurrence and ALC stays are much longer than normal hospital stays but typically not exceptionally long. It was surprising to learn this, as waiting placement patients then and now are often said to be common patients and ones with very long hospital stays (CIHI, 2009). This diversity in reality versus perception reveals some commonly held myths about aging and hospital utilization. Clearly, research evidence is important to counter inaccurate, pejorative, and unhelpful understandings of waiting placement. The reality is that only a small proportion of hospital patients and bed days in Alberta were being used by people who waited placement in hospital for a nursing home bed. Similar studies in other places are needed to determine if this is the case elsewhere, and to assess for trends. One United States study for instance established that the nursing home entry rate nationally was declining (Ness, Ahmed, & Aronow, 2004).

The qualitative findings revealed hospital stays consisted primarily of indefinite waiting after the ALC designation. This phase of the study was confined, however, to the small proportion of waiting-placement patients who were not actively dying or otherwise too ill and thus incompetent to provide informed consent and be interviewed. This phase of the study was therefore oriented to the people who would be the most impacted by a protracted, indeterminate, and highly significant wait. All of the participants knew they were not returning home but instead would go to live in a nursing home. Previous research has established that moving into a nursing home is highly stressful (Heliker & Scholler-Jaquish, 2006; McClendon, Smythe, & Neundorfer, 2006). The current study indicates that the stress of moving occurs in advance of the move, with this stress triggered by the realization that a move will occur and at some point in the unknown future whether they have agreed to it or not. As such, the findings of this study expand upon current evidence-based knowledge of the effects of relocation on older persons.

As waiting placement in hospital will undoubtedly continue to occur with population aging and ongoing healthcare limitations for restoring the health of all patients, it is important to consider how this transitional period of time can be improved. Research is needed to identify and test options, such as some emerging practices to help older people prepare for this move, and then wait for this move and move with less distress (McClendon et al., 2006). Research in particular should determine if being directly involved in the decision to relocate is helpful. Furthermore, research should determine if visiting one or more nursing homes will reduce fears of moving there, or if having a choice about the nursing home that they will move to will help ensure successful relocations. Counseling may also be indicated to help them accept this move, a matter that requires research to determine the most efficacious counseling for this highly significant move (Heliker & Scholler-Jaquish). Research is also needed to determine how much and what types of daily home care would be required to enable a return to home to wait placement there.

Research and action are also required for the finding of loneliness or social isolation in hospital. Socially-isolated and lonely people have been revealed previously as those who live at home alone with few if any social contacts or meaningful relationships (Nicolas & Nicholson, 2009). However, the current study shows ALC patients are socially isolated in hospital, and they experience loneliness and social isolation as a result of few visitors and busy nurses who understandably prioritize their time and care efforts to the acutely-ill patients.

The lack of visitors is also understandable, but highly regrettable. Not only are family and friends important for relieving or preventing boredom and feelings of loneliness, but familiar faces have long been identified as helpful for preventing and assisting recovery from illnesses, including acute delirium (Wilson et al., 2010). The studied waiting placement patients were in hospitals far from their homes or home communities, with visitors having to travel to see them. Many would have family members and friends who, like them, are advanced in age; and some will have outlived most of their family members and friends. Some did not marry or have children if married, and thus have few possible immediate family visitors (Kasper, Pezzin, & Rice, 2010). Others will no doubt have children or family members who are working, busy with other family commitments, and/or live far away (Kasper et al., 2010). Close proximity has already been established as a major factor for enabling frequent visits by family members (Yamamoto-Mitani, Aneshenset, & Levy-Storms, 2002). It is of concern that the socio-demographic circumstances that reduce visitor availability now will likely become more commonplace in the future with accelerating population aging, aging among seniors, and various already established marital, fertility, and migration trends (Kinsella & He, 2008).

The finding that is perhaps the most significant to highlight is that the hospital was revealed as a bleak living environment for waiting placement patients. This bleakness is not entirely surprising, as acute care hospitals were designed and are operated primarily for the purpose of diagnosing and treating acute illnesses (Sheps et al., 2000). Efficiency, cost-effectiveness, and infection control are guiding care principles there. Personal items are thus not encouraged as they could be lost or damaged, or could contribute to hospital-acquired infections, and with the number effectively limited then by small bedside tables and few other places to put them. Easy-to-change hospital-issue pajamas were used instead of personal sleepwear or street clothing. Not only was depersonalization thus clearly evident, but these waiting-placement patients did not appear to have been recognized as having any unique or special care needs. These needs were not only physical; but rehabilitative, social, and emotional in nature. This care gap needs to be considered in light of the requirement that waiting-placement patients in Canada must pay a nursing home accommodation fee (approximately $50) for each day that they wait in hospital for placement in a nursing home, yet they are not privy to the usual amenities of nursing homes. Although nursing homes are often criticized for low staffing, nursing homes are highly regulated in Canada. Most offer a wide range of programs and services to enhance quality of life, and these programs and services can potentially increase not only well-being but longevity (Wilson & Truman, 2004). The bleakness of hospitals as a temporary home or living space for ALC patients is also in stark contrast to the deliberately created home-like environment of nursing homes. It is important to note that some hospitals are establishing units that are designed specifically for waiting placement patients with the usual amenities of nursing homes present (Rowe, 2002). These specialized waiting placement units are controversial, however, as they do not reduce the number of persons waiting placement in hospital.

Other findings were concerning and should be addressed. Patient’s rights policies in hospitals may be needed to ensure waiting placement patients are involved whenever possible in placement decisions. Greater understanding and direct support are also likely required to help patients accept this life-changing decision and have their fears of moving to a nursing home reduced. Visits to the nursing home that they will eventually move into may therefore be helpful. In-hospital services or programs to prevent boredom and social isolation are also needed. More physical rehabilitation is critical for preventing deterioration from inactivity and to increase their recovery from the illness that necessitated this hospitalization. Advocacy also appears to be needed in relation to an expansion of home care services so some or many waiting placement patients can return home to wait for placement there (Beland et al., 2006; Caris-Verhallen & Kerkstra, 2001; Davey et al, 2005; Gaugler et al., 2000). It is becoming more and more evident that supportive community-based services for older dependent persons and their family caregivers are needed now and with additional ones needed in the future to keep pace with population aging (Fjelltun et al., 2009; Kinsella & He, 2008; Gaugler et al.; Ness et al., 2004).

## 5. Conclusions

This study sought evidence on the hospital utilization of waiting placement patients and insight into the lived experience of waiting in hospital for placement in a nursing home. The findings of this study highlight the importance of evidence to combat myths and other commonly held viewpoints about aging and waiting placement. Waiting placement was found to be an almost insignificant hospital utilization concern. However, and most significantly, the findings of this study indicate hospitals are unsuitable for waiting placement; with remedies to address waiting placement care deficits in hospitals needing to be planned and implemented. Alternative places to wait are perhaps a better option, such as the home with enhanced home care services so the person can safely wait there. At the very least, the findings should alert healthcare professionals, managers, researchers, and others to the many possible negative social, psychological, and physical impacts of this significant wait on elderly persons. As this was a study confined to two Canadian hospitals, hospital managers and healthcare professionals elsewhere are urged to assess their own hospital programs and services to determine if they meet the physical, social, and emotional care needs of patients who find they are waiting in hospital for placement in a nursing home.

## Figures and Tables

**Appendix:**
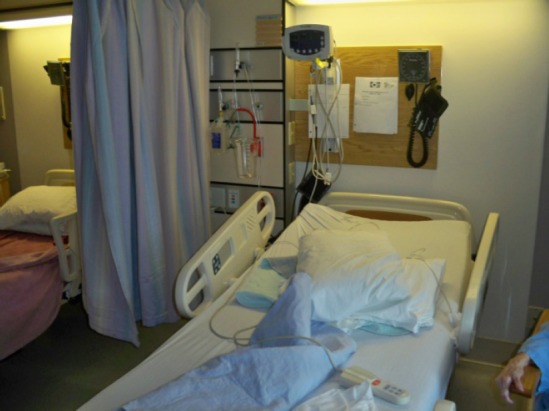
Two living space photographs
